# The tumor suppressor gene *KCTD11*^*REN *^is regulated by Sp1 and methylation and its expression is reduced in tumors

**DOI:** 10.1186/1476-4598-9-172

**Published:** 2010-06-30

**Authors:** M Michela Mancarelli, Francesca Zazzeroni, Lucia Ciccocioppo, Daria Capece, Agnese Po, Simona Murgo, Raffaello Di Camillo, Christian Rinaldi, Elisabetta Ferretti, Alberto Gulino, Edoardo Alesse

**Affiliations:** 1 Department of Experimental Medicine, University of L'Aquila, L'Aquila, 67100, Italy; 2 Department of Experimental Medicine, University "La Sapienza" of Rome, Rome 00161, Italy; 3 Neuromed Institute, Pozzilli, Italy

## Abstract

A hallmark of several human cancers is loss of heterozygosity (LOH) of chromosome 17p13. The same chromosomal region is also frequently hypermethylated in cancer. Although loss of 17p13 has been often associated with *p53 *genetic alteration or Hypermethylated in Cancer 1 (*HIC1*) gene hypermethylation, other tumor suppressor genes (TSGs) located in this region have critical roles in tumorigenesis. A novel TSG mapping on human chromosome 17p13.2 is *KCTD11*^*REN *^(*KCTD11*). We have recently demonstrated that KCTD11 expression is frequently lost in human medulloblastoma (MB), in part by LOH and in part by uncharacterized epigenetic events. Using a panel of human 177 tumor samples and their normal matching samples representing 18 different types of cancer, we show here that the down-regulation of KCTD11 protein level is a specific and a diffusely common event in tumorigenesis. Additionally, in order to characterize the regulatory regions in *KCTD11 *promoter, we identified a CpG island and several Sp1 binding sites on this promoter, and demonstrated that Sp1 transcription factor and DNA methylation contribute, at least in part, to regulate KCTD11 expression. Our findings identify *KCTD11 *as a widely down-regulated gene in human cancers, and provide a basis to understand how its expression might be deregulated in tumor cells.

## Findings

TSGs often locate at chromosomal regions, which are frequently deleted and/or methylated in tumors. High levels of 17p13 somatic alterations have been showed in several tumors, distal and independent of the *p53 *locus [1-4].

Our group has identified *KCTD11 *as an immediate-early gene induced by neurogenic signals [5] and encoding a novel adaptor of Cullin3 ubiquitin E3 ligase complex targeting Histone Deacetylase 1 [6]. Importantly, *KCTD11 *is a novel TSG that inhibits cell growth and is mapping on human chromosome 17p13.2, whose expression is frequently lost in human MB [4].

**Figure 1:**
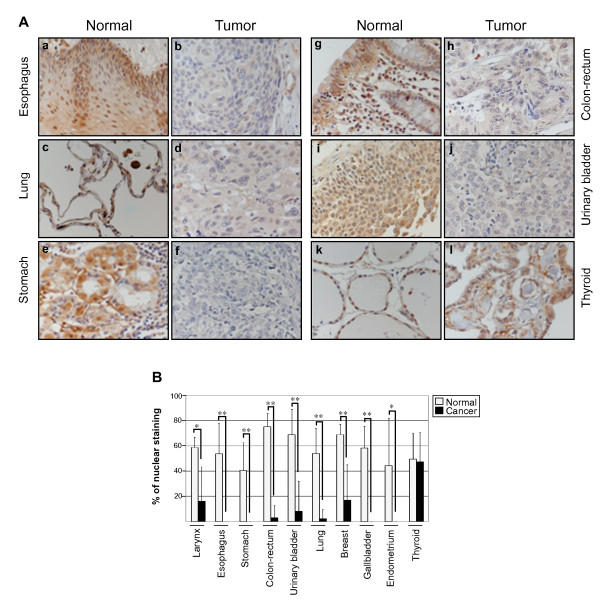
**KCTD11 is down-regulated in several cancers**. **(A) **Representative images of KCTD11 immunohistochemistry (IHC) of esophagus (**a**, **b**), lung (**c**, **d**), stomach (**e**, **f**), colon-rectum (**g**, **h**), urinary bladder (**i**, **j**) and thyroid (**k**, **l**) (40x magnification). Tissue arrays (Super Bio Chips; cat. n. MA, MAN, MB, MBN, MC, MCN; http://www.tissue-array.com/ver3/index.php) were incubated with 1 mg/ml affinity-purified rabbit polyclonal anti-KCTD11 antibody, as previously described [12]. **(B) **Graphic representation of KCTD11 IHC analysis of tissue arrays. Normal and cancer tissues were analyzed counting nuclear staining as percent point. 10 high power fields (hps 40x) were counted for each sample. Stromal and inflammatory cells were not selected for nuclear staining counting. The significance of differences between normal (N) and tumor (T) tissues means was estimated using Student's t-test. (* indicates p < 0.005; ** indicates p < 0.001). Larinx: N n = 5, T n = 7; Esophagus: N n = 9, T n = 10; Stomach: N n = 13, T n = 10; Colon-rectum: N n = 12, T n = 10; Urinary bladder: N n = 5, T n = 9; Lung: N n = 10, T n = 10; Breast: N n = 4, T n = 10; Gallbladder: N n = 8, T n = 6; Endometrium: N n = 7, T n = 10; Thyroid: N n = 10, T n = 10. n: number of cases for each group.

To analyze whether the down-regulation of *KCTD11 *represents a specific feature of MB, as well to other cancers, we performed a wide screening for KCTD11 expression, analyzing 177 human tumor samples and 177 normal matching samples, representing 18 different cancer types. Normal tissues, including larynx, esophagus, stomach, colon-rectum, urinary bladder, lung, breast, gallbladder and endometrium, exhibited a nuclear KCTD11 positive immunohistochemical staining between 40 to 78% (Fig. 1B), whereas the matching tumor samples showed a significant reduction of 0 to 18% of nuclear KCTD11 staining (Fig. 1A and 1B). Reduced KCTD11 expression was not observed in thyroid and kidney tumor tissues vs normal suggesting a tumorigenic specific role of KCTD11 for the above mentioned tissues (Fig. 1A and 1B and data not shown). Moreover KCTD11 was undetected both in normal and cancer tissues from liver, lymph-node and exocrine pancreas (data not shown). Together, these findings clearly indicated that selective tissues expressing KCTD11 have down-regulated this gene during tumorigenesis.

**Figure 2:**
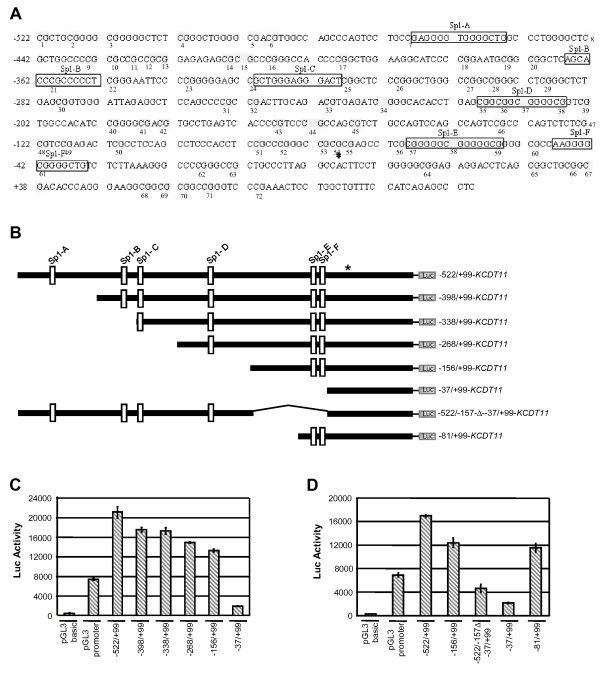
**Basal transcriptional activity of human *KCTD11 *promoter is depending on Sp1 TF**. **(A) **Human *KCTD11 *promoter was identified by Promoter Inspector http://www.genomatix.de/. Strong matches for Sp1 transcription factor binding sites are boxed. The asterisk indicates the TSS. The CpG dinucleotides are grey highlighted and numbered. (**B**) Schematic representation of Luc reporter constructs driven by human full-length *KCTD11 *promoter or deletion mutants. Constructs were generated by PCR using genomic DNA from peripheral blood lymphocytes as template and specific primers [additional file 2] and subcloned in pGL3 basic vector. (**C-D**) Luciferase assays showing the transcriptional activity of full-length or deletion mutants of *KCTD11 *promoter. 293T HEK (human embryo kidney) cell line was cultured in DMEM supplemented with 10% FCS, 2 mM L-glutamine (Sigma), 100 U/ml penicillin and 100 μg/ml streptomycin (Sigma). 2 μg of *KCTD11*-Luc reporter constructs were transfected using Lipofectamine 2000 (Invitrogen). After 24 hrs, the cells were harvested and firefly luciferase activity was assayed using the Firelite Dual Luminescence Reporter Gene Assay System kit (Perkin-Elmer) normalized to Renilla luciferase activity. Each experiment has been done in triplicates. Values are the means ± S.D.

To understand the transcriptional regulation of *KCTD11*, we identified and analyzed the promoter region. Human *KCTD11 *proximal promoter is a 623 bp region (Fig. 2A). It turned out to be a TATA- and CAAT-less promoter. The transcription start site (TSS) was previously identified [4] (Fig. 2A). Using the TRANSFACT software, we identified six putative binding sites for Sp1 transcription factor (TF), two of them located closely to the TSS (see Fig. 2A, Sp1-E and Sp1-F). Sp1 is a well-characterized transcriptional activator [7,8]. It is essential for proper expression of a large variety of genes involved in development, cell growth regulation and cancer [9,10]. Moreover, Sp1 is responsible for recruiting TATA-binding protein and fixing the TSS at TATAless promoters [9,11]. Thus, the Sp1-E and Sp1-F binding sites on *KCTD11 *promoter (Fig. 2A) are likely to be responsible for assembling of the basal transcription complex.

To understand the regulation of *KCTD11 *promoter by Sp1, we analyzed the transcriptional activity of full-length and various deleted forms of *KCTD11 *promoter (Fig. 2B-D). Surprisingly, full-length *KCTD11 *promoter showed a strong basal transcriptional activity (see Fig. 2C, -522/+99), as the luciferase activity was 2.5 folds higher than that of pGL3-promoter, containing the strong SV40 promoter. Although slightly decreased, *KCTD11 *promoter activity was not drastically dropped down by deletion of the Sp1-A, Sp1-B, Sp1-C and Sp1-D sites, whereas it resulted strongly dependent on Sp1-E and Sp1-F sites (Fig. 2B-C). Importantly, the interval between Sp1-B and Sp1-C sites is too short to allow Sp1 to bind both sites, and this might explain why -398/+99 (containing Sp1-B and Sp1-C) and -338/+99 (lacking Sp1-B) mutants showed the same transactivation ability. Similar results have been obtained by transfecting *KCTD11 *promoter constructs in other cell lines [additional file 1]. To verify the importance of Sp1-E and Sp1-F binding sites for *KCTD11 *promoter activity, a full-length *KCTD11 *promoter construct bearing a deletion -156/-38 - the region containing the Sp1-E and Sp1-F - was generated (Fig. 2B, -522/-157Δ-37/+99). The deletion of Sp1-E and Sp1-F sites strongly affected the ability of *KCTD11 *promoter to drive transcription (Fig. 2D, compare -522/-157Δ-37/+99 to -522/+99). Most likely, Sp1 binds only to one of these two sites, considering the short interval between Sp1-E and Sp1-F sites. To exclude that the Sp1-E upstream region -156/-82, which was also deleted in the -522/-157Δ-37/+99 construct, was relevant to the promoter activity, we further generated and tested an -81/+99 construct (Fig. 2B), demonstrating that this region did not have any effect on *KCTD11 *transcriptional activity (Fig. 2D, compare - 156/+99 and -81/+99).

**Figure 3:**
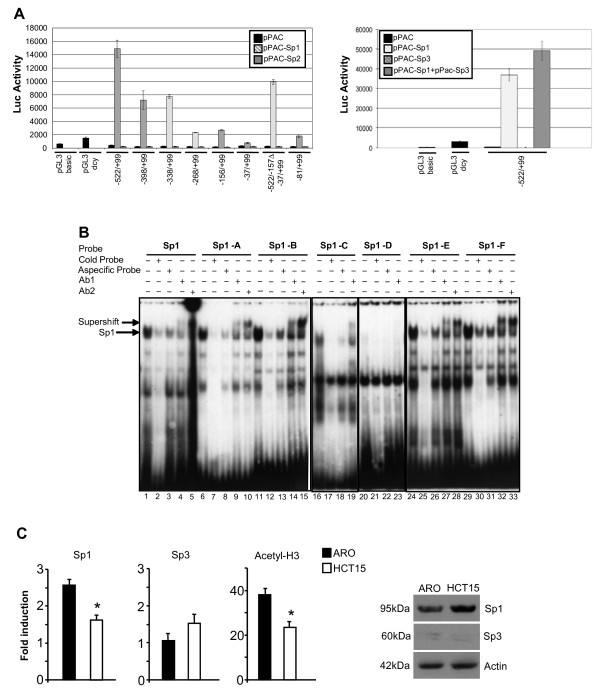
***KCTD11 *promoter is strongly activated by Sp1 TF**. (**A**) D. Mel-2 cells (Invitrogen) were cultured in Drosophila Serum-Free Medium (Gibco Life Technologies) plus18 mM L-glutamine (Sigma) at room temperature. *KCTD11*-Luc reporter constructs (1 μg) and pPac-LacZ (200 ng) were co-transfected along with 1 μg of pPac empty vector, pPac-Sp1 or pPac-Sp2 (left panel) or pPac-Sp3 and pPac-Sp1+pPac-Sp3 (right panel) using Cellfectin (Invitrogen). After 48 h, the cells were harvested and the luciferase activity was assayed using the Single Luciferase Assay System (Promega), normalized to β-galactosidase activity using β-Galactosidase Enzyme Assay System (Promega). Each experiment has been done in triplicates. Values are the means ± S.D. (**B**) EMSAs showing the binding of Sp1 to *KCTD11 *promoter. Whole extracts were prepared from 293T HEK cells transfected with pCDNA-Sp1 (12 μg), using buffer C [16]. Cell extracts were incubated *in vitro *with ^32^P-labeled KCTD11-Sp1 probes (Sp1-A-F) or ^32^P-labeled canonical Sp1 probe [additional file 2]. Binding specificity was evaluated by competition with an excess (100x) of the cold probe or with a non-specific probe. For the supershift assays, the proteins were pre-incubated with two different anti-Sp1 antibodies (Santa-Cruz) at 4°C for 30 min. (**C**) ARO (thyroid cancer) and HCT15 (colon cancer) cell line were cultured, respectively, in RPMI-1640 and DMEM supplemented with 10% FCS. Chromatin Immunoprecipitations (ChIP) were performed by using the following antibodies: anti-Sp1 (1C6) X (sc-420; Santa Cruz Biotech), anti-Sp3 (F-7) X (sc-28305; Santa Cruz Biotech), anti-Acetyl-Histone3 (Cell Signaling). Eluted DNA has been analyzed with real-time q-PCR, normalized to GAPDH (left panels). Bars represent the mean of 3 independent experiments ± SD (*, p < 0.05, HCT15 versus ARO). Total protein levels of Sp1 and Sp3 were analyzed by Western blot (right panel).

Because of Sp1 protein is constitutively expressed TF [9], to assess specifically the role of Sp1 in the regulation of *KCTD11 *promoter, we co-transfected the *KCTD11 *promoter constructs (Fig. 2B) along with Sp1, Sp2 or Sp3 expression vectors into Drosophila Mel-2 cells (Fig. 3A), which provide a null background for Sp TFs activity [9]. In this cellular system, none of *KCTD11 *promoter constructs showed basal activity when co-transfected along with an empty vector, whereas Sp1 co-transfection strongly activated the promoter (Fig. 3A). Moreover, the functional significance of each Sp1 site in the *KCTD11 *promoter was evaluated. Sp1-A and Sp1-C resulted to drive *KCTD11 *promoter activity (Fig. 3A, compare -398/+99 + pPac-Sp1 *vs *-522/+99 + pPac-Sp1 and - 268/+99 + pPac-Sp1 *vs *-338/+99 + pPac-Sp1), whereas Sp1-B and Sp1-D did not (Fig. 3A, compare -338/+99 + pPac-Sp1 *vs *-398/+99 + pPac-Sp1 and -156/+99 + pPac-Sp1 *vs *-268/+99 + pPac-Sp1). As a control, deletion of all Sp-1 sites completely abrogated *KCTD11 *promoter activation by Sp1 (Fig. 3A, -37/+99 + pPac-Sp1 column).

As D. Mel-2 cells are null for Sp TFs, Sp1 should not be responsible for recruiting TATA-binding protein and fixing the TSS at TATA-less promoters in this cell context. In fact, full-length *KCTD11 *promoter lacking Sp1-E/Sp1-F was just slightly (30%) less active than the full-length promoter (Fig. 3A, -522/-157Δ-37/+99 + pPac-Sp1 *vs *-522/+99 + pPac-Sp1; see also Fig. 2D). Therefore we demonstrated that the activation of *KCTD11 *promoter was Sp1 specific; conversely the co-transfection with Sp2 or Sp3 expression vectors failed to induce *KCTD11 *promoter activity (Fig. 3A). In addition, Sp3 did not influence Sp1-driven *KCTD11 *transcription (Fig. 3A, left panel). Next, we demonstrated that Sp1 binds all the Sp1 binding sites of *KCTD11 *promoter, except for Sp1-D, by performing EMSAs (Fig. 3B).

Finally, we assessed the *in vivo *relevance of Sp1-mediated regulation of *KCTD11 *gene, performing ChIP-qPCR for Sp1, Sp3 and Acetyl-H3 on ARO cells (a thyroid cancer cell line, matching a tumor tissue sample in which KCTD11 is expressed; see Fig. 1) and HCT15 cells (a colon cancer cell line, matching a cancer type in which KCTD11 is inhibited; see Fig. 1). As shown in Fig. 3C, Sp1 binds to *KCTD11 *promoter much strongly in ARO cells than in HCT15 cells, confirming that Sp1 plays a pivotal role in regulating *KCTD11 *gene expression, *in vivo*. At contrary, ARO and HCT15 cell lines showed no differences in Sp3 binding to *KCTD11 *promoter. Importantly, Acetyl-H3 binds to *KCTD11 *promoter much strongly in ARO cells than in HCT15 cells, indicating that this promoter is more acetylated in ARO cells than in HCT15 cells.

These data demonstrated that Sp1 TF is necessary for the basal transcriptional activity of *KCTD11 *TATA-less promoter.

The Sp-regulated promoters are often associated with GC-rich regions of the genome known as CpG islands, which are important for gene regulation [12]. The presence of six Sp1 binding sites on *KCTD11 *promoter and the mapping of human *KCTD11 *at the highly methylated chromosomal region 17p13 prompted us to investigate the involvement of DNA methylation in *KCTD11 *regulation.

**Figure 4:**
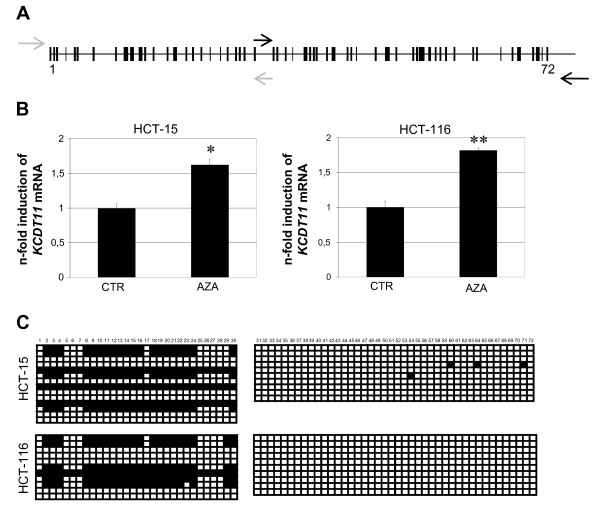
***KCTD11 *is silenced by methylation in HCT-15 and HCT-116 cell lines**. A CpG island, containing 72 CpG dinucleotides (indicated by the vertical lines) was identified from -522 bp to the +70 bp of *KCTD11 *promoter http://www.ebi.ac.uk/emboss/cpgplot/. (**B**-**C**) HCT-15 and HCT-116 colorectal carcinoma cells were cultured in DMEM supplemented with 10% FCS, 2 mM L-glutamine (Sigma), 100 U/ml penicillin and 100 μg/ml streptomycin (Sigma), and treated with 2 μM of Aza (Sigma) daily for 1 and 3 days, respectively. RNA was extracted by using Rneasy Mini Kit (Qiagen) and cDNA was generated from 1 μg of DNase I treated RNA using MuLV Reverse Trascriptase (Applied Biosystems). (**B**) *KCTD11 *mRNA level was analyzed by q-RT-PCR, normalized to GAPDH. The demethylating agent, Aza increased KCTD11 expression. Primers sequences are available in additional file 2. (* indicates p < 0.005; ** indicates p < 0.001). All measurements were performed in triplicates. Values are the means ± S.D. (**C) **Representation of methylated CpG found in *KCTD11 *promoter after bisulfite treatment of HCT-15 and HCT-116 cell lines. Closed and open boxes indicate methylated and unmethylated CpG sites, respectively. Horizontal lanes indicate methylation status of several PCR-amplified sodium bisulfite-treated HCT-15 and HCT-116 cDNA clones. PCRs were performed using two primer sets amplifying the CpG dinucleotides from 1 to 30 and from 31 to 72 [additional file 2; arrows on Fig. 4A]. PCR products were cloned into pCRII-TOPO-TA vectors (Invitrogen) and colonies screened by blue/white selection. Plasmid DNAs from at least ten colonies were sequenced and analyzed for C-T conversion.

We found a putative CpG island overlapping almost all *KCTD11 *promoter (-522 to +70), which contains 72 CpG dinucleotides (Fig. 2A; 4A). To evaluate whether methylation might regulate *KCTD11 *transcription, we investigated the effects of demethylation on KCTD11 expression, in two human colon cancer cell lines, HCT15 and HCT116. 5'-Aza-2'-deoxycytidine (Aza) treatment of these cells significantly increased *KCTD11 *mRNA level (Fig. 4B). It is worth to note that the induction of KCTD11 expression that we observed here with Aza is comparable with the induction by RA, NGF and EGF or during differentiation of cultured cerebellar GCPs observed in previous studies and there considered physiologically relevant [5,13]. We therefore investigated the methylation profile of *KCTD11 *promoter in HCT15 and HCT116 cell lines. DNAs from sodium bisulfite-treated HCT116 and HCT15 were amplified by PCR, subcloned and sequenced. Sequences showed that *KCTD11 *promoter is methylated in both HCT-15 and HCT-116 cell lines with a similar pattern (Fig. 4C). As control, DNAs from Aza and sodium bisulfite-treated HCT116 and HCT15 cells showed, as expected, C-T conversion of all cytosines (not shown). Together, these data clearly demonstrate a direct role of the methylation on *KCTD11 *transcriptional activity. Interestingly the regions of *KCTD11 *promoter resulting methylated are located mainly in the 5' region (Fig. 4C), in particular the region upstream of Sp1-A binding site (-517/-501 meaning CpGs 2 to 4), the region in between of Sp1-A and Sp1-C binding sites (-443/-331 meaning CpGs 8 to 24) and the region upstream of Sp1-D binding site (-279/-278 meaning CpG 30) (Fig. 2A, 4C).

Thus, the majority of the methylated CpGs in *KCTD11 *promoter are not located at Sp1 binding sites, but in the surrounding regions. To this regard, published reports have implicated Sp1 binding sites involved in the maintenance of the methylation-free status of the CpG islands [12,14,15]. Thus, the balance between DNA methylation and Sp1 binding to the promoter may represent a mechanism to regulate the tissue-specific expression pattern of KCTD11. It is noteworthy that the chromosomal region 17p13, where *KCTD11 *localized, is frequently hypermethylated in cancer. Therefore, the strong down-regulation of KCTD11 in cancers (Fig. 1) may be due to promoter hypermethylation. Of course, we cannot exclude that other mechanisms might play a role in the physio-pathological regulation of KCTD11 expression, such as inhibition of Sp1 activity by other proteins. In summary, we reported a dramatic down-expression of KCTD11 protein in several types of cancer. Importantly, we identified a CpG island and several Sp1 binding sites in the *KCTD11 *promoter region and further demonstrated that Sp1 transcription factor and DNA methylation contributed, at least in part, to regulate KCTD11 expression. These findings provide a basis to implicate that decreased expression of *KCTD11 *tumor suppressor gene is associated with cancers of specific tissue types.

## Abbreviations

KCTD11^REN^: Potassium Channel Tetramerization Domain 11; (Retinoic Acid-EGF-NGF); RA: Retinoic Acid; EGF: Epidermal Growth Factor; NGF: Nerve Growth Factor; LOH: loss of heterozygosity; TSG: Tumor Suppressor Gene; OVCA1: Ovarian Cancer-1; OVCA2: Ovarian Cancer-2; ABR: active BCR-related gene; HIC1: Hypermethylated in Cancer 1; MB: medulloblastoma; TSS: transcription start site; TF: transcription factor; Aza: 5'-Aza-2'-deoxycytidine; IHC: immunohistochemistry.

## Competing interests

The authors declare that they have no competing interests.

## Authors' contributions

MMM performed the experiments, conceived experiments, statistical analysis and helped to draft manuscripts. FZ drafted manuscript, conceived experiments, statistical analysis and coordinated the study. LC, RDC performed immunohistochemical staining. DC performed luciferase assay. AP performed chromatin immunoprecipitation experiments. SM participated in a part of the experiments. CR performed sequencing. EF provided some of the samples needed for this study. AG participated in design and coordination of this study and helped to draft the manuscript. EA provided financial support, participated design and coordination of this study and helped to draft the manuscript.

All authors read and approved the final manuscript.

## Supplementary Material

Additional file 1 ***KCTD11 *transcriptional activity in D283, TSU and HCT116 cell lines is comparable to the 293T *KCTD11 *transcriptional activity**. Luciferase assays showing the transcriptional activity of full-length or deletion mutants of *KCTD11 *promoter. D283 (medulloblastoma cell line) was cultured in Eagle's Minimum Essential Medium and TSU (prostate carcinoma cell line) was cultured in RPMI-1640 supplemented with 10% FCS, 2 mM L-glutamine (Sigma), 100 U/ml penicillin and 100 μg/ml streptomycin (Sigma); HCT116 was cultured as previously described in Figure 4. 2 μg of *KCTD11*-Luc reporter constructs were transfected using Lipofectamine 2000 (Invitrogen). After 24 hrs, the cells were harvested and firefly luciferase activity was assayed using the Firelite Dual Luminescence Reporter Gene Assay System kit (Perkin-Elmer) normalized to Renilla luciferase activity. Each experiment has been done in triplicates. Values are the means ± S.D. Click here for file

Additional file 2 **Table - Primer sequences for cloning *KCTD11 *promoter constructs, for q-PCR, for EMSA Sp1 canonic and Sp1-*KCTD11 *promoter probes, for CHIP-q-PCR and for methylation study**. Click here for file
