# Supplementary material for: The tumor suppressor gene KCTD11REN is regulated by Sp1 and methylation and its expression is reduced in tumors
Source: Mol Cancer. 2010 Jun 30;9:172. doi: 10.1186/1476-4598-9-172 (PMC2913982; doi:10.1186/1476-4598-9-172)
Supplement: Additional file 1 — KCTD11 transcriptional activity in D283, TSU and HCT116 cell lines is comparable to the 293T KCTD11 transcriptional activity. Luciferase assays showing the transcriptional activity of full-length or deletion mutants of KCTD11 promoter. D283 (medulloblastoma cell line) was cultured in Eagle's Minimum Essential Medium and TSU (prostate carcinoma cell line) was cultured in RPMI-1640 supplemented with 10% FCS, 2 mM L-glutamine (Sigma), 100 U/ml penicillin and 100 μg/ml streptomycin (Sigma); HCT116 was cultured as previously described in Figure 4. 2 μg of KCTD11-Luc reporter constructs were transfected using Lipofectamine 2000 (Invitrogen). After 24 hrs, the cells were harvested and firefly luciferase activity was assayed using the Firelite Dual Luminescence Reporter Gene Assay System kit (Perkin-Elmer) normalized to Renilla luciferase activity. Each experiment has been done in triplicates. Values are the means ± S.D. [file 1476-4598-9-172-S1.PPT]

## Slide 1
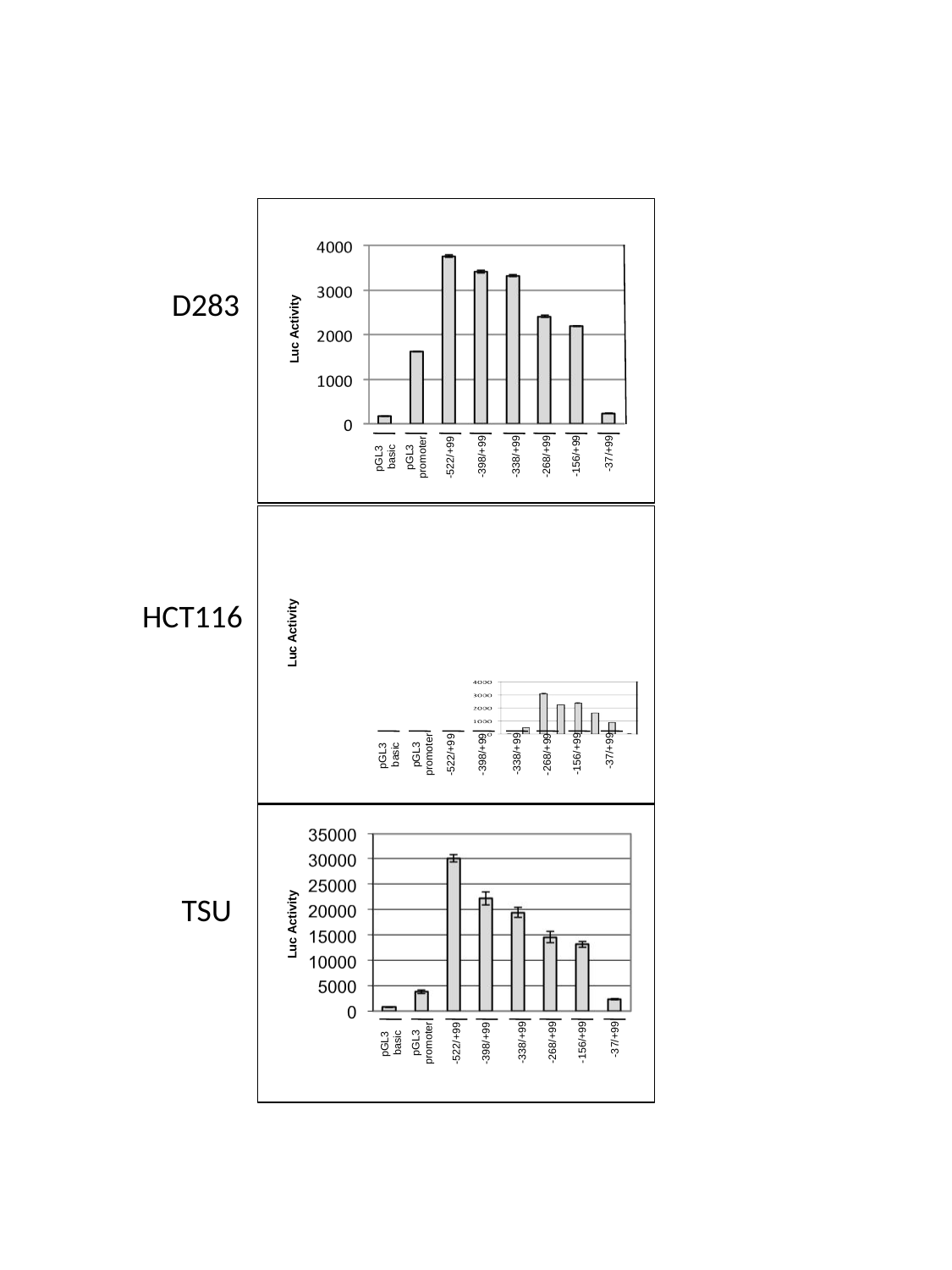

D283
Luc Activity
-398/+99
-338/+99
-268/+99
-37/+99
-522/+99
-156/+99
pGL3
promoter
pGL3
 basic
HCT116
Luc Activity
-398/+99
-338/+99
-268/+99
-37/+99
-522/+99
-156/+99
pGL3
promoter
pGL3
 basic
TSU
Luc Activity
-398/+99
-338/+99
-268/+99
-37/+99
-522/+99
-156/+99
pGL3
promoter
pGL3
 basic
